# First Report of the Local Spread of Vancomycin-Resistant Enterococci Ascribed to the Interspecies Transmission of a *vanA* Gene Cluster-Carrying Linear Plasmid

**DOI:** 10.1128/mSphere.00102-20

**Published:** 2020-04-08

**Authors:** Yusuke Hashimoto, Izumi Kita, Masato Suzuki, Hidetada Hirakawa, Hirofumi Ohtaki, Haruyoshi Tomita

**Affiliations:** a Department of Bacteriology, Gunma University Graduate School of Medicine, Maebashi, Gunma, Japan; b Department of Medical Technology, Kansai University of Health Sciences, Kumatori, Osaka, Japan; c Antimicrobial Resistance Research Center, National Institute of Infectious Diseases, Higashimurayama, Tokyo, Japan; d Laboratory of Bacterial Drug Resistance, Gunma University Graduate School of Medicine, Maebashi, Gunma, Japan; University of Nebraska Medical Center

**Keywords:** vancomycin-resistant enterococci, conjugative linear plasmid, local spread, interspecies transmission

## Abstract

Vancomycin-resistant enterococci pose a threat in the clinical setting and have been linked to hospital outbreaks worldwide. In 2017, a local spread of VanA-type vancomycin-resistant enterococci (VRE) occurred in Japan, and 25 enterococcal isolates, including 14 Enterococcus faecium, 8 E. raffinosus, and 3 E. casseliflavus isolates, were identified from four inpatients. Molecular analysis of the multispecies of VanA-type VRE revealed the involvement of both the dissemination of clonally related VRE strains between patients and the horizontal transfer of plasmids harboring the *vanA* gene cluster between *Enterococcus* spp. Pulsed-field gel electrophoresis showed that the plasmid DNAs without S1 nuclease treatment were able to migrate into the gel, suggesting that the topology of the plasmid was linear. Whole-genome sequencing revealed that this plasmid, designated pELF2, was 108,102 bp long and encoded multiple antimicrobial resistance genes, including *ermA* and *ant*(9). The amino acid sequences of putative replication- and transfer-related genes were highly conserved between pELF2 and pELF1, the latter of which was the first identified enterococcal conjugative linear plasmid. On comparing the genomic structure, pELF2 showed the presence of a backbone similar to that of pELF1, especially with respect to the nucleotide sequences of both terminal ends, indicating a hybrid-type linear plasmid, possessing two different terminal structures. pELF2 possessed a broad host range and high conjugation frequencies for enterococci. The easy transfer of pELF2 to different *Enterococcus* spp. *in vitro* might explain this local spread of multiple species, highlighting the clinical threat from the spread of antimicrobial resistance by an enterococcal linear plasmid.

**IMPORTANCE** Increasing multidrug resistance, including vancomycin resistance, in enterococci is a major concern in clinical settings. Horizontal gene transfer, such as via plasmids, has been shown to play a crucial role in the acquisition of vancomycin resistance. Among vancomycin resistance types, the VanA type is one of the most prevalent, and outbreaks caused by VanA-type vancomycin-resistant enterococci (VRE) have occurred worldwide. Here, we describe an enterococcal linear plasmid responsible for multispecies local spread of VanA-type VRE. Such a study is important because although hospital outbreaks caused by mixed enterococcal species have been reported, this particular spread indicates plasmid transfer across species. This is a crucial finding because the high risk for such a spread of antimicrobial resistance calls for regular monitoring and surveillance.

## Abstract (precis)

Increasing multidrug resistance, including vancomycin resistance, in enterococci is a major concern in clinical settings. Horizontal gene transfer, such as via plasmids, has been shown to play a crucial role in the acquisition of vancomycin resistance. Among vancomycin resistance types, the VanA type is one of the most prevalent, and outbreaks caused by VanA-type vancomycin-resistant enterococci (VRE) have occurred worldwide. Here, we describe an enterococcal linear plasmid responsible for multispecies local spread of VanA-type VRE. Such a study is important because although hospital outbreaks caused by mixed enterococcal species have been reported, this particular spread indicates plasmid transfer across species. This is a crucial finding because the high risk for such a spread of antimicrobial resistance calls for regular monitoring and surveillance.

## INTRODUCTION

Enterococci are normal inhabitants of the human intestinal tract and are typical nosocomial pathogens, with more than 40 species having been described thus far (1). Among the various *Enterococcus* spp., clinical infections are mainly caused by Enterococcus faecalis and E. faecium (2). In adult health care-associated infections in the United States, E. faecalis and E. faecium are the fifth and eighth most commonly reported pathogens, respectively (3). Other *Enterococcus* spp., including E. raffinosus and E. casseliflavus, are considered low-virulence organisms; however, with regard to severe enterococcal infections, the incidence of non-*faecium* and non-*faecalis* enterococcal bacteremia has been gradually increasing (2, 4). These clinical isolates have been reported to be resistant to clinical drugs, including penicillin, aminoglycosides, and glycopeptides (5). The Japan Nosocomial Infections Surveillance (JANIS) program showed that the rates of resistance to penicillin G, ampicillin, erythromycin, and levofloxacin were more than 80% among E. faecium isolates (https://janis.mhlw.go.jp/english/index.asp). Vancomycin has been among the antibiotic drugs most commonly used against severe Gram-positive bacterial infections (6, 7). For over 30 years after the clinical use of vancomycin was approved, vancomycin resistance was not a concern; however, since the first isolation of vancomycin-resistant enterococci (VRE), the number of VRE strains has been increasing (8–10). This is alarming because of the limited treatment options for VRE infections. As of 2018, the proportion of VRE isolates identified in Japan was reported to be less than 1% (JANIS; https://janis.mhlw.go.jp/english/index.asp); however, sporadic outbreaks have been occurring every year.

Most VRE outbreaks worldwide have been caused by E. faecium and E. faecalis and occurred because of the dissemination of clonally related VRE strains between patients or the horizontal transfer of glycopeptide resistance genes mediated by mobile genetic elements, such as transposons or plasmids, between *Enterococcus* spp. (5, 11–13). Of these, plasmids are considered to have the more significant impact because they are more amenable to intraspecies or interspecies transmission with *Enterococcus* spp. (14). In general, interspecies transfer of plasmids of *Enterococcus* spp. is considered to be much less frequent than intraspecies transfer (15). Recently, we discovered a transferrable linear plasmid harboring the *vanA* and *vanM* gene clusters from E. faecium in Japan (16). This plasmid conferring vancomycin resistance was reported to show interspecies transferability in enterococci.

In 2017, local spread of VanA-type VRE strains occurred in a community hospital in Japan. Here, we describe this VanA-type VRE spread and attempt to identify the plasmid underlying this event.

## RESULTS AND DISCUSSION

### Description of the local spread of VanA-type VRE and patient characteristics.

**FIG 1:**
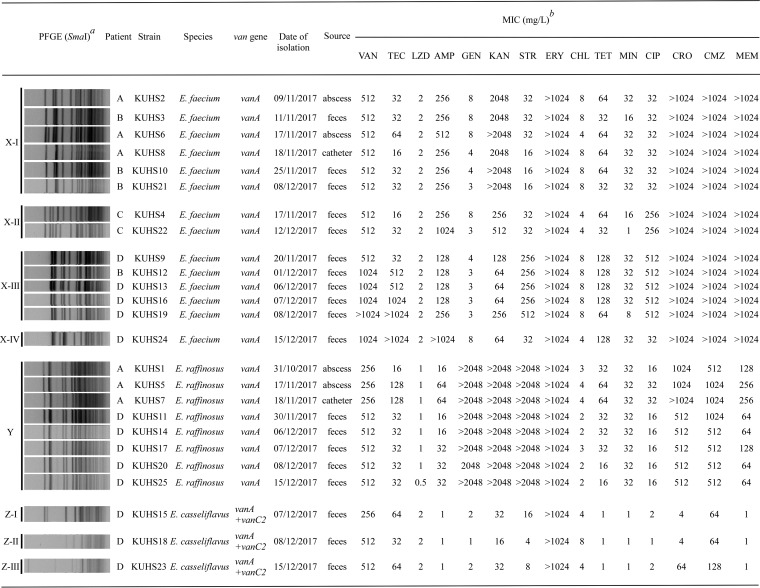
Bacterial strains used in this study and their pulsed-field gel electrophoresis (PFGE) patterns and drug susceptibilities. The superscript italic “a” indicates results of PFGE analysis of SmaI-digested DNA isolated from the locally spread strains. Pulse time varied from 5.3 to 34.9 s during the 20.0 h of electrophoresis. The superscript italic “b” indicates the following abbreviations and definitions: VAN, vancomycin; TEC, teicoplanin; LZD, linezolid; AMP, ampicillin; GEN, gentamicin; KAN, kanamycin; STR, streptomycin; ERY, erythromycin; CHL, chloramphenicol; TET, tetracycline; MIN, minocycline; CIP, ciprofloxacin; CRO, ceftriaxone; CMZ, cefmetazole; MEM, meropenem. To determine the MICs, E. raffinosus strains were grown for 48 h because their growth rate was low.

Index patient A was admitted to the hospital for the treatment of pneumonia. On day 31 of admission, a bloodstream infection with Staphylococcus caprae occurred for which vancomycin was administered intravenously for 18 days. The first vancomycin-resistant E. raffinosus isolate, designated KUHS1, was recovered from a sacral abscess on day 54 (see Fig. S1 in the supplemental material). Vancomycin-resistant E. faecium (KUHS2) was isolated from the same sacral abscess after a further 9 days; vancomycin-resistant E. faecium (KUHS6) and E. raffinosus (KUHS5) were also isolated after a further 8 days. In addition, vancomycin-resistant E. raffinosus (KUHS7) and E. faecium (KUHS8) were isolated from the central venous catheter. During this period, all patients present in the same room were screened using rectal swabs. The screening results revealed that among six patients, three (patients B to D) carried the vancomycin-resistant E. faecium strain in their feces. In the case of patient D, the presence of vancomycin-resistant E. raffinosus and E. casseliflavus was accompanied by that of vancomycin-resistant E. faecium. Regardless of frequent screening tests, vancomycin-resistant E. casseliflavus was isolated only from patient D. Unlike patient A, who exhibited the invasive VRE infection, no clinical signs of infection were detected in patients B to D, indicating that they were carriers. In total, 25 VRE isolates (14 E. faecium, 8 E. raffinosus, and 3 E. casseliflavus) were collected from four inpatients during a period of 2 months. All these isolates were determined to harbor the *vanA* gene by the use of a multiplex PCR assay (17). Data from JANIS revealed that the number of isolates of VRE in the prefecture, where the hospital is located, was low (vancomycin-resistant E. faecium, 1.7% [*n* = 75/4,420] in 2017; vancomycin-resistant E. faecalis, 0.0% [*n* = 3/9,499] in the same year). Furthermore, VRE isolates had not been identified from other inpatients for over 5 years before the isolation of KUHS1 in this hospital. These findings suggested the local spread of multispecies of VRE in the hospital during a short period of time. The MICs of antibiotics are shown in Fig. 1. Analyses of these MICs revealed that all the isolates exhibited high-level resistance to vancomycin. In addition, all of them showed resistance to erythromycin.

10.1128/mSphere.00102-20.1 FIG S1 Time course of isolation of multispecies vancomycin-resistant enterococci (VREs) from four patients. Dates of isolation of multispecies VREs from patients A to D are shown. Characters by themselves represent Enterococcus faecium isolates, characters on gray background represent Enterococcus raffinosus isolates, and characters in boxes represent Enterococcus casseliflavus isolates. Characters in brackets represent SmaI-PFGE patterns. N.D., not detected. Download FIG S1, TIF file, 0.05 MB. Copyright © 2020 Hashimoto et al. 2020 Hashimoto et al. This content is distributed under the terms of the Creative Commons Attribution 4.0 International license.

### Pulsed-field gel electrophoresis analyses of multispecies of VanA-type VRE.

To analyze the genetic correlations between these isolates, pulsed-field gel electrophoresis (PFGE) was performed (Fig. 1; see also Fig. S2) (16). On the basis of the patterns of SmaI-digested chromosomal DNA, isolates were categorized into three major groups. Accordingly, 14 E. faecium isolates were shown to belong to group X, which was divided into four subgroups (four subtypes, including X-I to X-IV). All E. raffinosus isolates belonged to the same group, the Y group, and three E. casseliflavus isolates belonged to group Z, which was divided into three subgroups (subtypes I to III). According to a guideline proposed previously by Tenover (18), the PFGE results showed that some of these isolates were indistinguishable, suggesting the occurrence of clonal dissemination of the VRE strains between the different patients. For example, regarding E. raffinosus in group Y, clonal isolates were identified in the comparisons between patients A and D. In addition, among the E. faecium strains in group X-I and group X-III, clonal VRE isolates were detected between patients A and B and patients B and D, respectively. However, the coexistence of isolates showing different PFGE patterns was also determined among E. faecium and E. casseliflavus isolates. For patient D, the number of isolated species of VRE increased over time during that short period (Fig. S1). Therefore, these findings implicating the involvement of genetically unrelated isolates indicated the horizontal transfer of glycopeptide resistance genes.

10.1128/mSphere.00102-20.2 FIG S2 Pulse-field gel electrophoresis (PFGE) analysis of SmaI-digested DNA of the locally spread strains. PFGE analysis of SmaI-digested DNA isolated from the strains was performed. Pulse times ranged from 5.3 to 34.9 s during the 20.0 h of electrophoresis. Download FIG S2, TIF file, 0.2 MB. Copyright © 2020 Hashimoto et al. 2020 Hashimoto et al. This content is distributed under the terms of the Creative Commons Attribution 4.0 International license.

### Molecular analyses of the plasmid carrying the *vanA* gene cluster.

**FIG 2:**
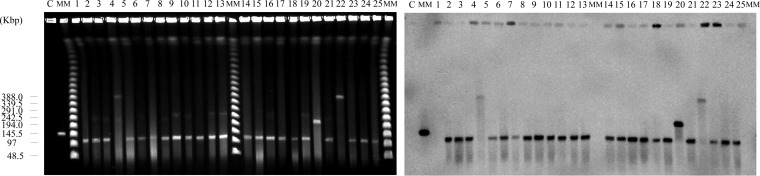
Pulsed-field gel electrophoresis (PFGE) analysis of S1 nuclease-untreated DNA and Southern blotting of the locally spread strains. PFGE analysis of S1 nuclease-untreated DNA (left) and Southern hybridization with a *vanA* gene probe (right). Lanes: C, AA708, a control strain harboring the linear plasmid pELF1; MM, Lambda Ladder PFG Marker (New England BioLabs); 1, KUHS1; 2, KUHS2; 3, KUHS3; 4, KUHS4; 5, KUHS5; 6, KUHS6; 7, KUHS7; 8, KUHS8; 9, KUHS9; 10, KUHS10; 11, KUHS11; 12, KUHS12; 13, KUHS13; 14, KUHS14; 15, KUHS15; 16, KUHS16; 17, KUHS17; 18, KUHS18; 19, KUHS19; 20, KUHS20; 21, KUHS21; 22, KUHS22; 23, KUHS23; 24, KUHS24; 25, KUHS25.

Because clonal dissemination of VRE strains alone cannot fully explain the PFGE results, we proceeded to confirm the plasmid content of these isolates. According to the results of PFGE performed with S1 nuclease as previously described (16), all these isolates carried several plasmids (Fig. S3). In particular, 22 of 25 isolates harbored an ∼110-kb plasmid, with the exceptions being KUHS4, KUHS20, and KUHS22. To confirm the plasmid topology, PFGE without S1 nuclease was performed (Fig. 2). The levels of electrophoretic mobility of these plasmids seen with or without S1 nuclease treatment were not different, indicating that they corresponded in a linear manner (Fig. 2; see also Fig. S3) (19). In contrast to the 22 isolates that seemed to carry the same linear plasmid, the size of each plasmid of KUHS4, KUHS20, and KUHS22 was observed to be larger and not consistent with that of each of the others; however, these plasmids also migrated into the PFGE gel without S1 nuclease treatment, suggesting that their topology was also linear (Fig. 2). Southern blotting using a *vanA* gene probe revealed that the *vanA* gene cluster was located on these linear plasmids (Fig. 2) (20), indicating that linear plasmid-mediated horizontal transfer had occurred between *Enterococcus* spp.

10.1128/mSphere.00102-20.3 FIG S3 Pulse-field gel electrophoresis (PFGE) analysis of S1 nuclease-treated DNA of the locally spread strains. PFGE analysis of S1 nuclease-treated DNA isolated from the strains was performed. Pulse times ranged from 5.3 to 66 s during the 19.5 h of electrophoresis. Lanes: MM, Lambda ladder PFG marker (New England BioLabs); 1, KUHS1; 2, KUHS2; 3, KUHS3; 4, KUHS4; 5, KUHS5; 6, KUHS6; 7, KUHS7; 8, KUHS8; 9, KUHS9; 10, KUHS10; 11, KUHS11; 12, KUHS12; 13, KUHS13; 14, KUHS14; 15, KUHS15; 16, KUHS16; 17, KUHS17; 18, KUHS18; 19, KUHS19; 20, KUHS20; 21, KUHS21; 22, KUHS22; 23, KUHS23; 24, KUHS24; 25, KUHS25; C, AA708, a control strain harboring linear plasmid pELF1. Download FIG S3, TIF file, 0.6 MB. Copyright © 2020 Hashimoto et al. 2020 Hashimoto et al. This content is distributed under the terms of the Creative Commons Attribution 4.0 International license.

### Whole-genome-sequencing-based analyses of the linear plasmid.

**FIG 3:**
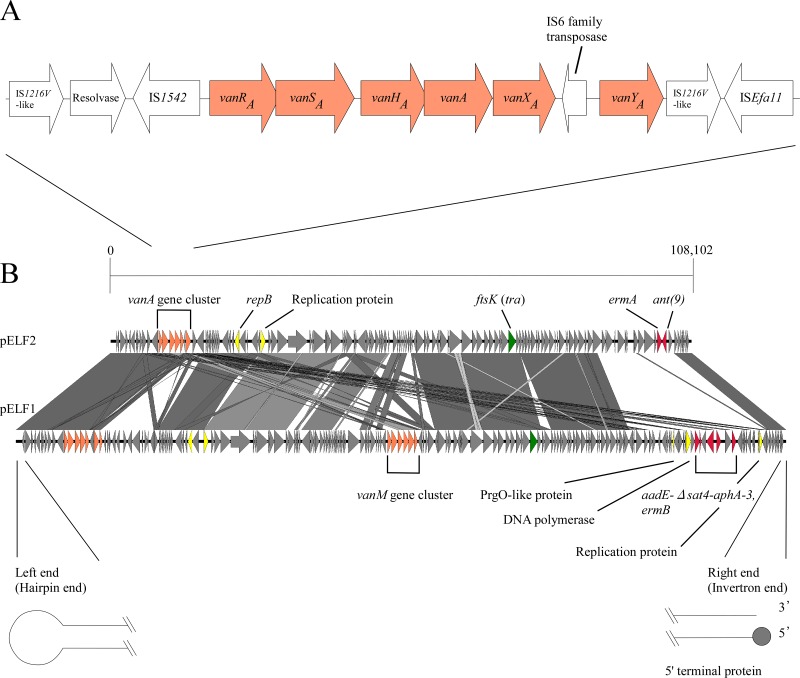
Schematics of the genetic structure of the *vanA* gene cluster (A) and pELF2 (B). The panels show the genetic structure of the *vanA* gene cluster carried on pELF2 (A) and a genomic comparison between pELF2 and pELF1 generated by Easyfig (B) (21). Gray lines connecting the nucleotide sequences of the two plasmids represent nucleotide identity of >67%. Arrows represent vancomycin resistance genes (orange), other drug resistance genes (red), putative replication-related genes (yellow), and putative transfer-related genes (green), respectively.

To investigate the genetic structure, whole-genome sequencing (WGS) of the E. faecium KUHS13 isolate was performed (see Table S1 in the supplemental material) using short-read Illumina MiniSeq (Illumina, San Diego, CA, USA) and long-read Nanopore MinION (Oxford Nanopore Technologies, Oxford, United Kingdom). The complete sequence of KUHS13 included the chromosome and three plasmids (Table S2). Two of the plasmids were circular and 253,148 and 6,258 bp long, respectively, whereas the third one (pELF2) was presumed to be linear and 108,102 bp long. According to the annotation, the pELF2 plasmid carried the *vanA* gene cluster, consistent with the results of our Southern blotting. The *vanA* gene cluster was carried on the Tn*1546*-like family. In the *vanA* gene cluster, a lack of the *vanZ* gene was identified; however, all other genes comprising the *vanA* gene cluster were intact (Fig. 3A). In addition, pELF2 also harbored drug resistance genes, including *ermA* and *ant*(9). Interestingly, compared to pELF1, the first identified enterococcal conjugative linear plasmid, pELF2, was found to harbor a similar genomic backbone (Fig. 3B) (21). Notably, the nucleotide sequences of both ends were shown to be highly conserved between pELF2 and pELF1, suggesting that pELF2 was a linear plasmid possessing two different terminal structures (Fig. S4) (16). The left end of pELF2 was presumed to be a hairpin structure harboring a 5′-TATA-3′ loop. In contrast, the right end contained abundant palindromic sequences forming secondary loop structures as shown by mfold analysis (Fig. S5) (22). Putative *repB* and *ftsK* genes were also carried on pELF2 (Fig. 3B). Notably, FtsK was considered to be a translocator protein involved in plasmid transfer (23, 24). The amino acid sequences of RepB and FtsK from pELF2 displayed 92.2% and 98% identities with the corresponding proteins from pELF1, respectively, indicating that the replication and transfer mechanisms of pELF2 were highly similar to those of pELF1.

10.1128/mSphere.00102-20.4 FIG S4 Nucleotide sequence alignment of each end of the pELF2 and pELF1 linear plasmids. The nucleotide sequence alignments of the left (A) and right (B) ends of pELF2 and pELF1 are shown. The box indicates putative hairpin sequences of the left end (5′-TATA-3′). Black boxes indicate single nucleotide variations between pELF2 and pELF1. Download FIG S4, TIF file, 0.7 MB. Copyright © 2020 Hashimoto et al. 2020 Hashimoto et al. This content is distributed under the terms of the Creative Commons Attribution 4.0 International license.

10.1128/mSphere.00102-20.5 FIG S5 Secondary structure of the right-end nucleotide sequence of pELF2. (A) The secondary structure of the 280-nucleotide sequence of the 3′ right end of pELF2 was predicted using mfold software (ΔG = −52.31 kcal/mol) (A) (22). (B) The 280-nucleotide sequence of the 3′ right end of pELF2 is shown. Arrows and Roman numerals indicate palindromic sequences. Boxes indicate predicted loop-structure sequences of each palindromic sequence. Download FIG S5, TIF file, 0.3 MB. Copyright © 2020 Hashimoto et al. 2020 Hashimoto et al. This content is distributed under the terms of the Creative Commons Attribution 4.0 International license.

10.1128/mSphere.00102-20.6 TABLE S1 Whole-genome sequence statistics. The superscript italic “a” indicates reads that were checked using Nanoplot (ver. 1.25.0) (36). The superscript italic “b” indicates reads that were checked using FastQC (ver. 0.11.8) (37). Download Table S1, XLSX file, 0.01 MB. Copyright © 2020 Hashimoto et al. 2020 Hashimoto et al. This content is distributed under the terms of the Creative Commons Attribution 4.0 International license.

10.1128/mSphere.00102-20.7 TABLE S2 Detailed information of WGS of KUHS13. The superscript italic “a” indicates that WGS was performed using Illumina Miniseq and Nanopore Minion and data were assembled using Canu (v1.8) and refined with Racon (v1.3.1.1) and Pilon (v1.20.1) (30–32). Download Table S2, XLSX file, 0.01 MB. Copyright © 2020 Hashimoto et al. 2020 Hashimoto et al. This content is distributed under the terms of the Creative Commons Attribution 4.0 International license.

### Transferability of linear plasmid pELF2 to different *Enterococcus* spp.

To confirm the horizontal plasmid transfer *in vitro*, conjugation experiments were performed. The pELF2 plasmid was easily transferred to E. faecium, E. faecalis, E. hirae, and E. casseliflavus by both filter and broth mating. The observed frequencies were 10^−3^ to 10^−5^ per donor cell in filter mating and 10^−6^ per donor cell in broth mating (Table S3). The transfer frequency seemed to be relatively high (15). The resulting transconjugants exhibited resistance to glycopeptides and erythromycin, consistent with the results of our WGS analysis (Fig. 3; see also Table S4). To investigate the possibility of the presence of an existing plasmid-like prophage, we prepared and collected the supernatant from a mitomycin C-treated KUHS13 culture (25). However, transfer of pELF2 could not be detected in recipient strains incubated with this supernatant.

10.1128/mSphere.00102-20.8 TABLE S3 Transfer frequency of pELF2 between KUHS13 and recipients strains. The superscript italic “a” indicates the frequencies were estimated from the CFU ratio of transconjugant strains to donor strains. The values represent means of three independent experiments with standard errors. Download Table S3, XLSX file, 0.01 MB. Copyright © 2020 Hashimoto et al. 2020 Hashimoto et al. This content is distributed under the terms of the Creative Commons Attribution 4.0 International license.

10.1128/mSphere.00102-20.9 TABLE S4 MICs of various antibiotic drugs. The superscript italic “a” indicates the following abbreviations and definitions: VAN, vancomycin; TEC, teicoplanin; LZD, linezolid; AMP, ampicillin; GEN, gentamicin; STR, streptomycin; KAN, kanamycin; ERY, erythromycin; CHL, chloramphenicol; TET, tetracycline; MIN, minocycline; CIP, ciprofloxacin; CRO, ceftriaxone; CMZ, cefmetazole; MEM, meropenem. The superscript italic “b” indicates the corresponding transconjugants that were obtained by filter mating with the donor KUHS13 strain. The superscript italic “c” indicates instances in which the strain was used as a recipient strain for the conjugation experiment. Download Table S4, XLSX file, 0.01 MB. Copyright © 2020 Hashimoto et al. 2020 Hashimoto et al. This content is distributed under the terms of the Creative Commons Attribution 4.0 International license.

### Conclusion.

Here, we described the interspecies parallel dissemination of clonal VRE strains and the linear plasmid carrying the *vanA* gene cluster during a local spread in a single hospital in Japan. To our knowledge, this is the first report of a local spread of VRE as a result of the transmission of a linear plasmid.

Hospital outbreaks caused by mixed enterococcal species have already been reported, but this kind of local spread seems to be rare (13, 26) and might explainable partially by the presence of plasmid-transfer barriers across species. In general, the rate of plasmid transfer between different enterococcal species was low (15); however, our PFGE results showed interspecies transfer of pELF2, consistent with results of conjugation experiments performed *in vitro*. Our results highlight the clinical importance of the enterococcal conjugative linear plasmid. The linear plasmids of KUHS4, KUHS20, and KUHS22 were found to be longer than the pELF2 plasmid. This finding suggests concurrent evolution of enterococcal linear plasmids during the local spread.

The clinical threat of non-*faecium* and non-*faecalis* enterococci has been often underestimated because they do not cause frequent infections (27, 28); however, non-*faecium* and non-*faecalis* enterococci have the potential to cause outbreaks of infection (29). Moreover, they might become important reservoirs of vancomycin resistance genes (13). As described above, pELF2 appears to possess a broad host range in enterococci. Our study also emphasized the importance of the surveillance of non-*faecium* and non-*faecalis* VRE. Because of the lack of information and epidemiological data concerning enterococcal linear plasmids and the absence of knowledge regarding the molecular mechanisms of plasmid transfer and plasmid replication, further intensive studies will be needed to clarify these points.

## MATERIALS AND METHODS

### Strains and culture media.

Bacterial strains isolated and used in this study are shown in Fig. 1. Screened stool cultures were inoculated onto BD vancomycin-resistant enterococcal selective agar (Becton, Dickinson and Company, Franklin Lakes, NJ, USA) at 35°C. After purification was performed at least twice, these strains were subjected to further investigations. Enterococci were grown in Todd-Hewitt broth (THB; Difco, Detroit, MI, USA) at 37°C.

### Antimicrobial susceptibility test.

After overnight culture, each strain was grown in Mueller-Hinton broth (MHB; Nissui, Tokyo, Japan), and the cultures were diluted 1:100 with fresh MHB. Approximately 5 × 10^5^ cells were spotted onto a series of Mueller-Hinton agar plates (Eiken, Tokyo, Japan) containing the appropriate test drugs. The MICs of antibiotics were determined using the agar-dilution method according to the CLSI guidelines (http://clsi.org/). To determine MICs, E. raffinosus strains were grown for 48 h owing to their low growth rate.

### Pulsed-field gel electrophoresis.

PFGE analysis was performed as previously described (16). Briefly, 1% agarose plugs containing embedded enterococcal strains were digested with lysozyme solution (Roche Diagnostics K.K., Minneapolis, MN, USA) (10 mg/ml) at 37°C for 6 h, followed by digestion of proteinase K (Merck Millipore, Darmstadt, Germany) solution (60 mAnson units/ml) at 50°C for 48 h. For analysis of genetic relatedness, enterococcal DNA embedded in agarose plugs was digested using SmaI (New England Biolabs, Ipswich, MA, USA) at 25°C for 12 h and then subjected to PFGE using a Chef Mapper system (Bio-Rad, Hercules, CA, USA). Pulse times ranged from 5.3 to 34.9 s during the 20.0 h of electrophoresis. For analysis of plasmid contents, enterococcal DNA embedded in agarose plugs was digested using S1 nuclease (Promega, San Luis Obispo, CA, USA) at 37°C for 20 min, and then subjected to PFGE using CHEFF MAPPER. Pulse time varied from 5.3 to 66 s during the 19.5 h of electrophoresis.

### Southern blot hybridization.

To determine the topology of plasmids and the location of *vanA*, enterococcal DNA without S1 nuclease digestion was prepared and subjected to PFGE, as described above. Southern blot hybridization was performed with a digoxigenin-based nonradioisotope system (Boehringer GmbH, Mannheim, Germany) according to the manufacturer’s protocol.

### Whole-genome sequencing.

Hybrid assembly was performed using short-read Illumina MiniSeq (Illumina) and long-read Nanopore MinION (Oxford Nanopore Technologies). WGS of strain KUHS13 was performed using a MiniSeq system (Illumina) with a High Output reagent kit (300 cycles). The library for sequencing (insertion size, 500 to 900 bp) was prepared using a Nextera XT DNA library prep kit (Illumina). On the other hand, the DNA library for Nanopore MinION was prepared using a rapid barcoding kit (SQK RBK-004; Oxford Nanopore Technologies) from total DNAs extracted using a Qiagen Genomic-tip 20/G or Gentra Puregene yeast/bacteria kit (Qiagen, Hilden, Germany) and then sequenced on a MinION flow cell (R9.4.1). WGS statistics are shown in Table S1 in the supplemental material. Raw data sets from the Nanopore MinION assay were submitted to Porechop (v0.2.3). The reads were assembled *de novo* using Canu (v1.8) (30). After the data were trimmed from Canu, they were polished with Racon (v1.3.1.1) and Pilon (v1.20.1) (31, 32). The nucleotide sequence of the left end of the linear plasmid (pELF2) was further checked by Sanger sequencing. DFAST and RAST were used to obtain the annotation (33, 34).

### Conjugation experiments and transfer frequency.

Conjugation experiments were performed as previously described (16, 35). KUHS13 was used as the donor strain, and FA2-2 (E. faecalis), BM4105RF (E. faecium), ATCC 9790RF (E. hirae), and KT06RF (E. casseliflavus) were used as the recipient strains (Fig. 1; see also Table S3). Briefly, for filter mating, the overnight cultures of donor and recipient strains were diluted 1:50 with fresh THB and then incubated at 37°C until the end of the exponential phase. Each 100 μl of the donor or the recipient strain was mixed with 5 ml THB. The mixture was collected on a 0.22-μm-pore-size nitrocellulose membrane filter (Merck) and then incubated on a THB plate for 5 h. After the membrane filter was washed with 1 ml of THB by vortex mixing, the mating mixture was plated on a selective THB plate containing vancomycin (12 mg/liter), rifampin (25 mg/liter), and fusidic acid (25 mg/liter) and was incubated at 37°C for 24 to 48 h.

For broth mating, the preculture was prepared as described above. Fifty microliters of the donor culture and 450 μl of the recipient culture were mixed in 4.5 ml of THB. The mixture of donor and recipient cells was incubated at 37°C with gentle shaking for 3 h and then plated on a selective THB plate, as described above. Each transfer frequency was estimated from the CFU ratio of transconjugant strains to donor strains.

### Accession number(s).

The data set for this study (the assembled sequence of KUHS13 strain) can be found in the DNA Data Bank of Japan database (DDBJ) (https://www.ddbj.nig.ac.jp/) under accession numbers AP022341, AP022342, AP022343, and AP022344. The Illumina reads and Nanopore reads of KUHS13 strain have been deposited in DDBJ under accession number DRA009515.
