# Supplementary figures and images for: First Report of the Local Spread of Vancomycin-Resistant Enterococci Ascribed to the Interspecies Transmission of a vanA Gene Cluster-Carrying Linear Plasmid
Source: mSphere. 2020 Apr 8;5(2):e00102-20. doi: 10.1128/mSphere.00102-20 (PMC7142295; doi:10.1128/mSphere.00102-20)

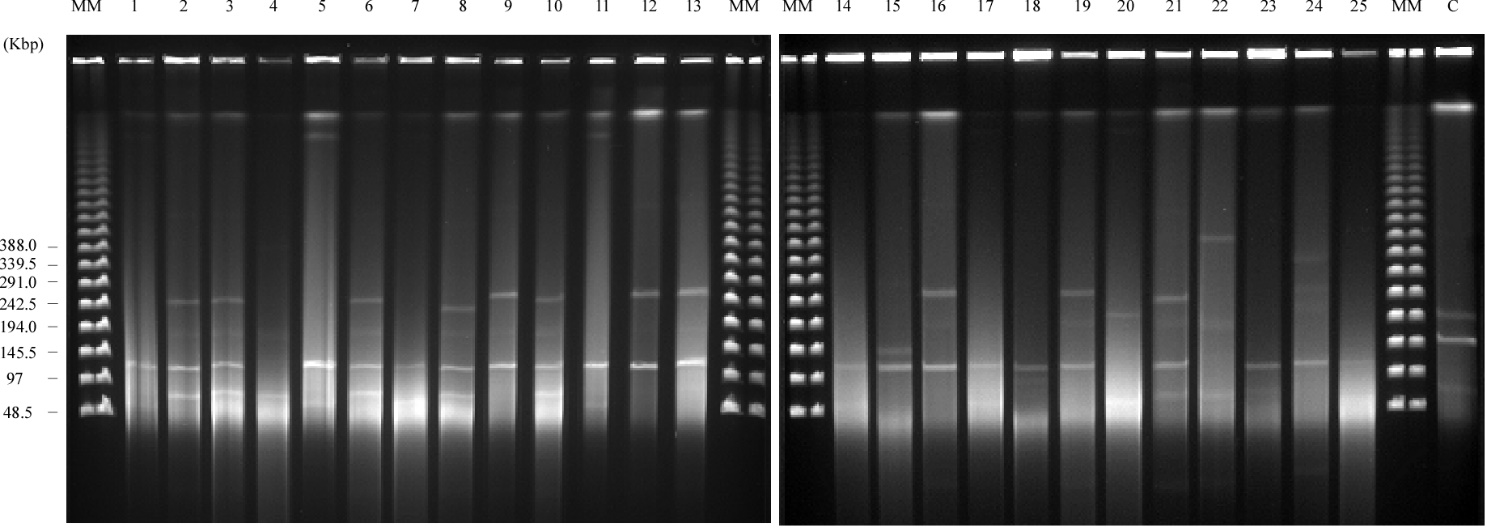

Supplement: FIG S3 [file mSphere.00102-20-sf003.tif]

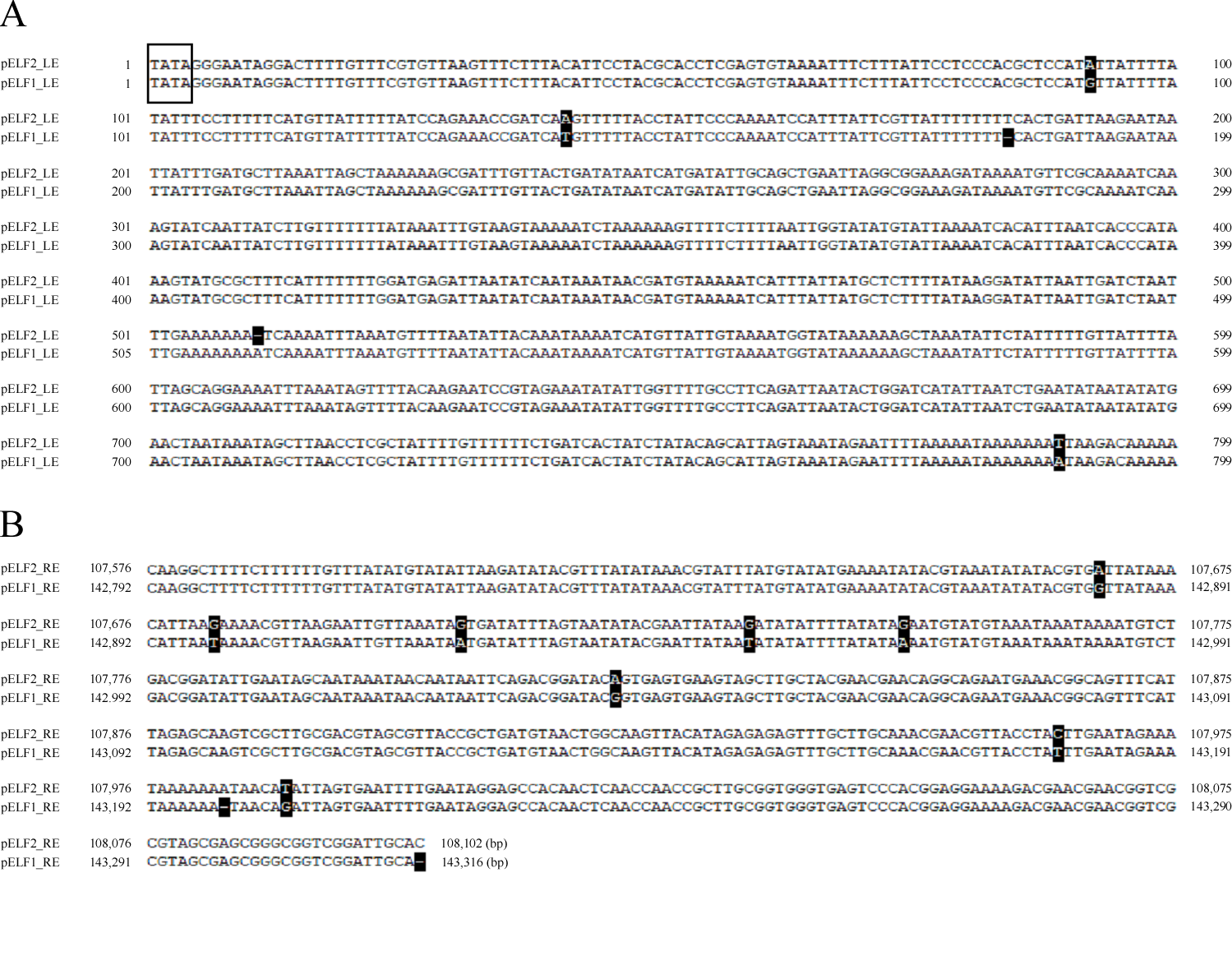

Supplement: FIG S4 [file mSphere.00102-20-sf004.tif]

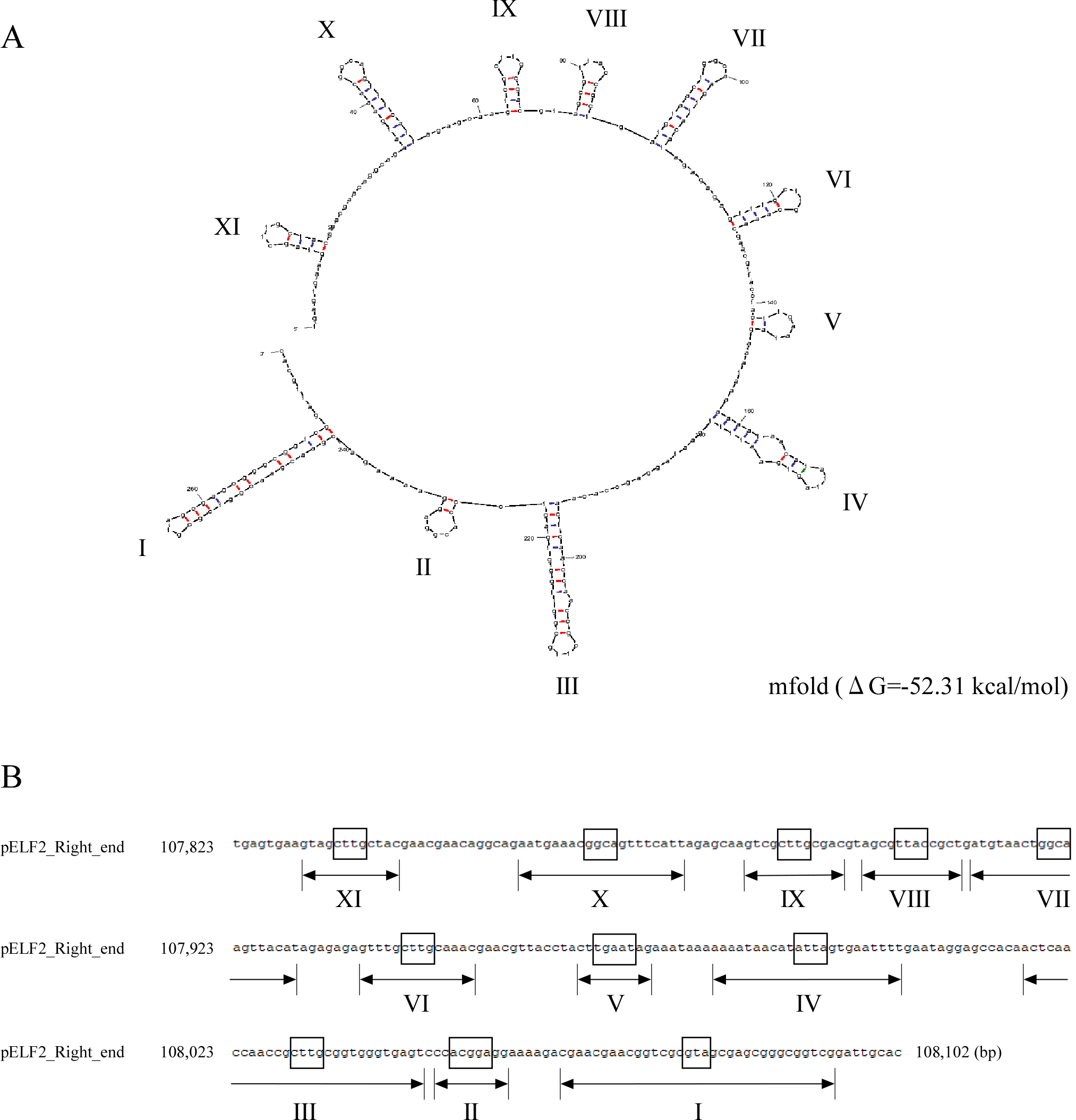

Supplement: FIG S5 [file mSphere.00102-20-sf005.tif]
